# HrpL Regulon of Bacterial Pathogen of Woody Host *Pseudomonas savastanoi* pv. *savastanoi* NCPPB 3335

**DOI:** 10.3390/microorganisms9071447

**Published:** 2021-07-05

**Authors:** Alba Moreno-Pérez, Cayo Ramos, Luis Rodríguez-Moreno

**Affiliations:** 1 Área de Genética, Facultad de Ciencias, Campus Teatinos s/n, Universidad de Málaga, E-29010 Málaga, Spain; albamp@uma.es; 2 Departamento de Microbiología y Protección de Cultivos, Instituto de Hortofruticultura Subtropical y Mediterránea «La Mayora», Extensión Campus de Teatinos, Universidad de Málaga-Consejo Superior de Investigaciones Científicas (IHSM-UMA-CSIC), E-29010 Málaga, Spain

**Keywords:** *P. savastanoi* pv. *savastanoi* (Psv), type III secretion system (T3SS), HrpL regulon, RNA-seq analysis, *hrp*-box prediction, virulence factors

## Abstract

The *Pseudomonas savastanoi* species comprises a group of phytopathogenic bacteria that cause symptoms of disease in woody hosts. This is mediated by the rapid activation of a pool of virulence factors that suppress host defences and hijack the host’s metabolism to the pathogen’s benefit. The *hrpL* gene encodes an essential transcriptional regulator of virulence functions, including the type III secretion system (T3SS), in pathogenic bacteria. Here, we analyzed the contribution of HrpL to the virulence of four pathovars (pv.) of *P. savastanoi* isolated from different woody hosts (oleander, ash, broom, and dipladenia) and characterized the HrpL regulon of *P. savastanoi* pv. *savastanoi* NCPPB 3335 using two approaches: whole transcriptome sequencing (RNA-seq) and the bioinformatic prediction of candidate genes containing an *hrp*-box. Pathogenicity tests carried out for the *P. savastanoi* pvs. showed that HrpL was essential for symptom development in both non-host and host plants. The RNA-seq analysis of the HrpL regulon in *P. savastanoi* revealed a total of 53 deregulated genes, 49 of which were downregulated in the Δ*hrpL* mutant. Bioinformatic prediction resulted in the identification of 50 putative genes containing an *hrp*-box, 16 of which were shared with genes previously identified by RNA-seq. Although most of the genes regulated by HrpL belonged to the T3SS, we also identified some genes regulated by HrpL that could encode potential virulence factors in *P. savastanoi*.

## 1. Introduction

The type III secretion system (T3SS) is considered one of the most relevant virulence mechanisms in animal and plant pathogenic bacteria. The T3SS is a complex membrane-embedded nanomachine through which Gram-negative bacteria translocate a set of proteins, known as type III effectors (T3Es), into the cytoplasm of host cells [1,2,3,4,5,6,7]. Structurally, the injectosome is made up of more than 20 proteins and is the most complex secretion system in bacteria [8]. From a functional point of view, the translocation of T3Es contributes to perturbing host cellular functions to facilitate bacterial survival and host colonization [6,9,10,11,12,13]. The T3SS is essential for *Pseudomonas syringae* pathogens to thrive in plant tissues. Although the evolution of the T3SS remains controversial, phylogenetic analysis through amino acid sequence comparison suggests that the T3SS first emerged in plant pathogens as an evolutionary adaptation of the flagellar export apparatus [4].

The *P. syringae* species complex is considered one of the most relevant phytopathogenic bacteria worldwide, due to its capacity to infect the phyllosphere and cause disease in a diverse range of cultivated, ornamental, and wild plants [14,15]. The complex comprises 13 phylogroups (PGs) encompassing 15 *Pseudomonas* species [16,17] that can be divided into about 65 pathovars (pv.) defined by their host ranges [18]. Except for some naturally occurring non-pathogenic *P. syringae* strains that lack the canonical T3SS [19,20,21], most of the strains included in the *P. syringae* complex require a functional T3SS for pathogenesis in susceptible plants [4,22,23]. 

The T3SS in *P. syringae* is encoded and regulated by the products of the hypersensitive response and pathogenicity (*hrp*) and hypersensitive response and conserved (*hrc*) gene clusters, which are included in a tripartite pathogenicity island together with other genes that encode accessory and conserved T3Es [24,25]. After translocation into the host cytoplasm, T3Es subvert host cellular functions, facilitating bacterial survival and host colonization [6,9,10,11,12,13]. The recognition of T3Es or their activity by the plant immune system, through resistance proteins or other mechanisms, induces the host’s hypersensitive response (HR), a localized plant cell death response that limits bacterial growth [26]. For this reason, the T3SS and its T3E repertoire have been recognized as the main determinants of host specificity in *P. syringae* [27,28,29].

The transcriptional regulation of T3SS’ structural components and their associated T3E repertoire in *P. syringae* is dependent on the HrpL regulator, which encodes an alternate sigma (σ) factor that recognizes a conserved promoter sequence (GGAACC-N15/16-CCACNNA), known as the *hrp*-box [30]. HrpL’s expression depends on the σ^54^ factor RpoN and two transcriptional activators, HrpR and HrpS, which work as heterodimers and cooperate with σ^54^ to promote the expression of *hrpL* [31,32]. Recent studies have clearly shown that the signaling pathways and molecular mechanisms involved in T3SS regulation in *P. syringae* are a complex, intricate network [33] involving dozens of regulatory proteins [8,34], second messenger molecules such as c-di-GMP [35], and variations in the physicochemical conditions during host colonization [36]. 

*Pseudomonas savastanoi* belongs to the PG3 group of the *P. syringae* complex; the unique PG includes knot-producing bacteria in woody hosts [16,37,38]. *P. savastanoi* comprises five pathovars that cause diseases in woody plants: pv. *savastanoi* (Psv, isolated from olive), pv. *nerii* (Psn, isolated from oleander), pv. *fraxini* (Psf, isolated from ash), pv. *retacarpa* (Psr, isolated from broom), and pv. *mandevillae* (Psm, isolated from dipladenia) [37,39]. In the Psv and Psn strains, the functionality of the T3SS has been shown to be essential for knot formation in the respective hosts and the induction of a characteristic HR in resistant hosts [37,40,41,42,43]. Recent comparative genomics analysis of strains belonging to these five pathovars identified the codification of highly conserved canonical T3SSs in strains of Psf, Psm, and Psr. However, their functionality and roles in pathogenesis in these three pathovars have not yet been established [39,44,45]. Furthermore, and as previously reported for Psv NCPPB 3335 [46], an additional T3SS resembling that found in Rhizobiaceae was also found in the other pathovars [39,44]. The relevance of T3SS regulation by HrpL in *P. savastanoi* is evidenced by the inability to induce knot formation in olive plants and to induce HR in tobacco plants by using a Δ*hrpL* mutant of the model Psv strain NCPPB 3335 [47]. Furthermore, pathovar-specific regulation of the T3SS and its T3E genes has been identified in Psv, Psn, and Psf, suggesting a possible role in host range, depending on the physiological conditions found in the apoplast, or the extracellular space of the host plant tissues [45].

The global regulation of transcription by HrpL in the *P. syringae* complex has been approached using microarrays or RNA-seq strategies. However, the HrpL regulon has only been defined in *P. syringae* strains isolated from herbaceous hosts [48,49], and no data are available for strains isolated from woody hosts. Here, we constructed HrpL mutants of model Psn, Psm, Psf, and Psr strains to study the role of this regulator in the pathogenicity of *P. savastanoi* strains isolated from other woody hosts. Then, we defined the HrpL regulon of Psv NCPPB 3335, the only *P. savastanoi* strain whose chromosome [44,46] and plasmids [50] have been fully sequenced. For this purpose, we used two approaches: (i) the comparative transcriptomic analysis (RNA-seq) of wild-type Psv NCPPB 3335 and its Δ*hrpL* mutant, and (ii) the bioinformatic prediction of *hrp*-box promoters in the genome of this strain. A comparison of the results obtained from these analyses with those previously reported for *P. syringae* strains isolated from herbaceous hosts allowed us to unravel novel HrpL-dependent genes that may play a role in the virulence of *P. savastanoi* and the interactions of bacterial pathogens with woody hosts.

## 2. Materials and Methods

### 2.1. Bacterial Strain, Plasmids, and Growth Conditions

The bacterial strains and plasmids used in this study are described in Tables S1 and S2, respectively. All the *P. savastanoi* strains were grown at 28 °C in lysogeny broth (LB) medium [51] without glucose and containing 0.5% NaCl, in King’s B (KB) medium [52] or in Super Optimal Broth (SOB) medium [53]. *Escherichia coli* strains were grown in LB medium at 37 °C. When required, the medium was supplemented with the following: for *P. savastanoi*: ampicillin (Ap) (400 μg/mL), kanamycin (Km) (7 μg/mL), nitrofurantoin (Nf) (25 μg/mL), and cycloheximide (Ch) (100 μg/mL); for *E. coli*: Ap (100 μg/mL) and km (50 μg/mL).

### 2.2. Construction of P. savastanoi Mutants

To construct the *hrpL* mutants, the complete gene was removed from model strains of Psn, Psm, Psf, and Psr. These mutants were constructed using the pIAC4-Km plasmid previously described [47] (Table S2), which contains a DNA fragment of approximately 1.2 kb corresponding to the 5′ and 3′ flanking regions of the Psv NCPPB 3335 *hrpL* gene and the *nptII* (Km)-resistance gene. This plasmid was electroporated into Psn *Psn23*, Psm Ph3, Psf NCPPB 1006, and Psr CECT 4861, as previously described [54]. The mutants were screened and verified as previously described [47]. Thereafter, the kanamycin gene was removed using the pFLP2 plasmid [55].

### 2.3. Plant Bioassays

The *Nicotiana tabacum* var. Newdel and *Solanum lycopersicum* var. MoneyMaker plants used in the HR assays were 3 months and 4–6 weeks old, respectively. The plants were grown with a photoperiod of 16 h of light and 8 h of darkness, with day/night temperatures of 26 and 22 °C, respectively. The leaves were infiltrated with bacterial suspensions in 10 mM MgCl_2_ (5 × 10^7^ to 1 × 10^8^ CFU/mL) of the Δ*hrpL* mutants using a blunt syringe. The generated symptoms were captured with a high-resolution digital camera (Nikon DXM 1200; Nikon Corporation, Tokyo, Japan) at 48 h post-inoculation.

The pathogenicity of the Δ*hrpL* mutants was analyzed for *Nerium oleander* plant accession “pink” (single pink flowers) supplied by Viveros Guzmán (Málaga, Spain), *Fraxinus excelsior* and *Retama sphaerocarpa* plants native to Valladolid and supplied by Viveros Fuenteamarga (Valladolid, Spain), or *Mandevilla* spp. var. red flowers supplied by New Plants Motril SA (Motril, Spain). Two plants per strain were inoculated, as previously described in [44]. The number of wound sites infected per plant varied between 10 and 12, depending on the size of the plant. 

### 2.4. Preparation of Samples for RNA-Seq Analysis

A pre-inoculum of 20 mL of the wild-type *P. savastanoi* pv. *savastanoi* NCPPB 3335 and its Δ*hrpL* mutant was grown overnight in KB medium at 28 °C. The cells were diluted in 2 cultures of 110 mL of fresh KB medium at an OD_600_ of 0.1 and incubated with agitation at 28 °C to an OD_600_ of 0.5 (approximately 5 × 10^7^ CFU/mL). Then 48 mL of each culture was pelleted; one of these was frozen, and the other was washed twice with 10 mM MgCl_2_ and resuspended in the same volume of Hrp-inducing medium [56]. After 6 h of incubation, each culture was divided into 6 samples of 8 mL and pelleted. For each strain, there were 2 biological replicates divided into 8 mL samples. A sample per biological replicate and strain was processed for RNA isolation using the RNAeasy Mini Kit (Qiagen, Hilden, Germany). Isolated total RNA was treated twice with a TURBO DNA-free Kit (Invitrogen, Carlsbad, CA, USA). The RNA concentration was determined spectrophotometrically, and its integrity was assessed by agarose gel electrophoresis. Before sequencing, we performed RT-qPCR on genes (*hrpA*, *avrPto1*, and *hopAO2*) whose expression was already known to be regulated by HrpL (Figure S1). This confirmed that the expression of those 3 genes was repressed in the RNA samples obtained from the *hrpL* mutants. Two independent RNA extractions (two biological replicates) from each strain were sent to the Ultrasequencing Service of the University of Malaga, where the necessary quality checks were carried out and RNA sequencing was performed. Sample quality control was performed with a LabChip RNA 6000 Pico (Agilent Technologies, Santa Clara, CA, USA). An Illumina Ribo-Zero Plus rRNA Depletion Kit (Illumina, San Diego, CA, USA) was used for the degradation of ribosomal RNA. The libraries were prepared with a TruSeq Stranded mRNA Kit (Illumina, San Diego, CA, USA), and sequencing was performed on an Illumina NextSeq550.

### 2.5. Sequence Mapping and Analysis

The RNA reads were analyzed by the Ultrasequencing Service of the University of Malaga. The Illumina adapters, lower-quality bases, and ribosomal sequences were removed using SeqTrimNext (https://rubygems.org/gems/seqtrimnext/versions/2.0.60, accessed on 1 June 2021). Quality control was performed with the FastQC software. Then, the reads were aligned to the complete sequence of the chromosome of Psv NCPPB 3335 (accession number: NZ_CP008742.1) and those of its 3 native plasmids (pPsv48A, FR820585.2; pPsv48B, FR820586.1; pPsv48C, FR820587.2) using *Bowtie* 2 (v. 2.2.9) [57]. Differential gene expression and transcript abundance were calculated using the Tuxedo Suite [58] with some modifications. Within this Suite, the Cufflinks program was used to estimate the aligned readings in the different transcripts and estimate their abundance. The fragments per kilobase per million mapped reads (FPKM) values were used to normalize and quantify gene expression. A false discovery rate (FDR) with a significance level of 0.05 (*q* value) and a minimum log_2_ (fold change) of ±0.5 was used to judge the significance of differences in gene expression. A graphical representation of the differential expression results was constructed using the cummeRbund package in R [59].

### 2.6. RT-qPCR Assays

For quantitative real-time PCR (RT-qPCR), DNA-free total RNA obtained as described in the previous section was used. In this procedure, 1 µg of DNA-free total RNA was retrotranscribed to cDNA using a cDNA iScript^TM^ cDNA synthesis kit (Bio-Rad, Hercules, CA, USA) and random hexamers. The RT-qPCR primers were designed with free online software according to the instructions previously described (Table S3) [60]. The primer efficiency tests, RT-qPCR, and confirmation of amplification reactions were assessed according to the criteria previously described [61]. Each reaction was carried out initially for 2 min at 95 °C, followed by 45 cycles of PCR (95 °C for 15 s and 59 °C for 30 s). The relative transcript abundance was calculated using the ΔΔ cycle-threshold (Ct) method [62]. The data obtained were normalized to the *gyrA* housekeeping gene and represented as fold change in expression compared with the expression of each gene in the wild-type strain. The relative expression ratio was calculated as the difference in the Ct of the gene of interest and *gyrA* (ΔCt = Ct*gen* of interest – Ct*gyrA*). One PCR cycle represents a twofold difference in template abundance; therefore, fold change values were calculated as 2^−ΔΔCt^, as previously described [63,64].

### 2.7. Prediction of HrpL-Dependent Genes

To search for HrpL-dependent genes, we used an ad hoc pipeline considering the presence of potential HrpL boxes (*hrp*-boxes) 500 nucleotides upstream of the start codon, as previously described [65]. To identify novel *hop* genes, we analyzed the N-terminal sequence features of the selected genes using EffectiveDB [66]. 

### 2.8. Bioinformatic Characterization of Identified Genes

A comparison was made of the genes identified in this study with the genes whose dependence on HrpL was previously demonstrated by RNA-seq in 6 strains of the *Pseudomonas syringae* complex isolated from herbaceous hosts: *P. syringae* pv. *tomato* (Pto) DC3000, *P. syringae* pv. *phaseolicola* (Pph) 1448A, *P. syringae* pv. *syringae* (Psy) B728A, *P. syringae* pv. *lachrymans* (Pla) 107, *P. syringae* pv. *japonica* (Pja) MAFF 301072, and *P. syringae* pv. *oryzae* (Por) 1_6 [48,49]. This comparison was made by blastp using Geneious 8.1.9 [67]. In addition, a more specific search looking for protein domains was carried out with Pfam [68] and HHPred [69].

## 3. Results

### 3.1. HrpL of P. savastanoi Pathovars Is Required for the Induction of Hypersensitive Response in Non-Susceptible Hosts and Symptom Development in Susceptible Hosts

**Figure 1:**
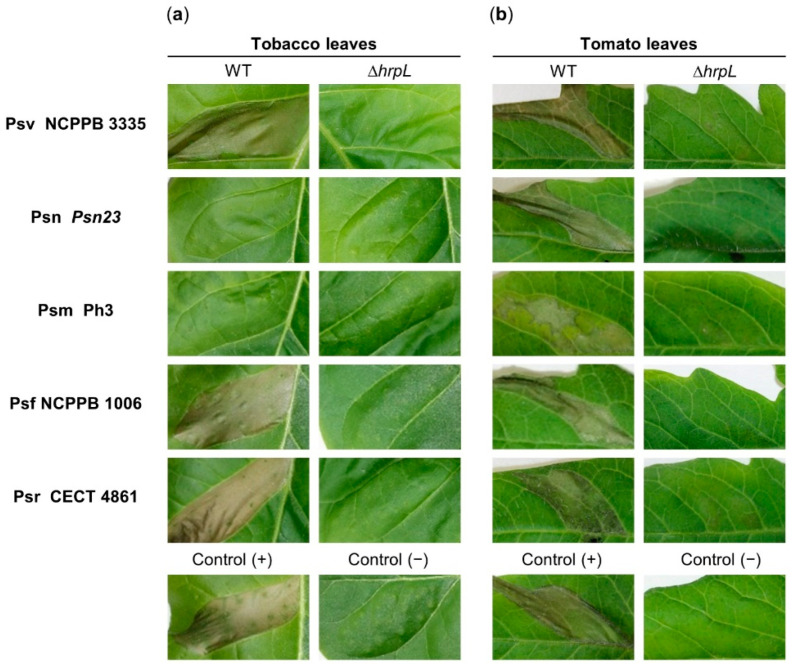
Hypersensitive response (HR) produced by wild-type (WT) strains of *P. savastanoi* pathovars and their ∆*hrpL* mutants in *Nicotiana tabacum* var. Newdel (tobacco) and *Solanum lycopersicum* var. Moneymaker (tomato) leaves. (**a**) HR symptoms induced on tobacco leaves following bacterial infiltration 24 h post-inoculation. Control (+), leaves inoculated with *Pseudomonas syringae* pv. *tabaci* CFBP 1621. (**b**) HR symptoms induced on tomato leaves after 24 h post-inoculation. Control (+), leaves inoculated with *Pseudomonas syringae* pv. *tomato* DC3000. Control (−), mock-infiltrated plants with 10 mM MgCl_2_.

The type III secretion system has been described as a key virulence factor in Psv [42,43,47]. To analyze the role of HrpL in the virulence of Psn, Psm, Psf, and Psr, we generated Δ*hrpL* mutants of Psn *Psn23*, Psm Ph3, Psf NCPPB 1006, and Psr CECT 4861. First, we analyzed the capacity of *P. savastanoi* strains to induce HR in tobacco plants, a non-susceptible host (Figure 1a). As previously reported, the infection of Psv NCPPB 3335, included as positive control, induced characteristic HR symptoms 24 h post-inoculation (Figure 1a). Out of the four remaining pathovars, Psf NCPPB 1006 and Psr CECT 4861 were also able to induce HR symptoms in tobacco leaves. However, no HR symptoms were observed on tobacco leaves infiltrated with wild-type Psn *Psn23* and Psm Ph3 after 24 h (Figure 1a). Based on this result, we carried out infiltration assays in tomato plants (Figure 1b). The same as the positive control, all the pathovars were able to induce HR symptoms on tomato leaves after 24 h. However, none of the Δ*hrpL* mutants were able to induce the formation of HR symptoms 24 h post-inoculation in either tobacco or tomato leaves (Figure 1a,b).

**Figure 2:**
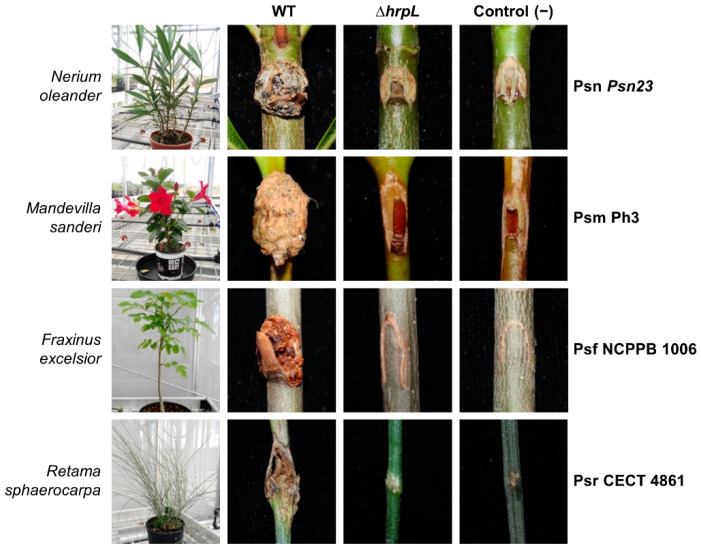
Symptoms induced in oleander, dipladenia, ash, and broom plants 90 days after inoculation with wild-type (WT) strains of *P. savastanoi* pathovars and their Δ*hrpL* mutants. Control (−), plants mock-inoculated with 10 mM MgCl_2_.

Besides its role in the development of HR symptoms in non-susceptible hosts, HrpL is also required for full symptom induction by Psv in olive plants [47]. To evaluate the contribution of HrpL to the virulence of the other four *P. savastanoi* pathovars, we carried out pathogenicity tests of Δ*hrpL* mutants in their respective host plants (Figure 2). As expected, wild-type Psn *Psn23*, Psm Ph3, Psf NCPPB 1006, and Psr CECT 4861 were able to induce tissue proliferation (overgrowth) at the inoculation points 90 days post-inoculation. By contrast, none of the inoculated Δ*hrpL* mutants showed tumor symptoms at inoculation points that were distinguishable from the mock control plants 90 days post-inoculation (Figure 2).

### 3.2. RNA-Seq Transcriptome Profiles for Psv NCPPB 3335 and Its ΔhrpL Mutant

Previous work reported that non-effector genes controlled by the HrpL regulon vary across the *P. syringae* phylogeny [48]. Out of the six *P. syringae* strains analyzed by Mucyn and collaborators [48], none was pathogenic in a non-herbaceous plant. Our Psv NCPPB 3335 reference strain [44,46] and its Δ*hrpL* mutant [47] were cultivated on Hrp-inducing medium [56], which simulates in planta apoplastic conditions, to obtain the total RNA. To compare transcript abundance, two biological replicates of each strain were subjected to Illumina RNA sequencing, and a total of 177.83 million (81.9 million from the wild-type and 95.8 million from the mutant strain) 100-bp paired-end reads was generated (Table S4).

The raw reads were trimmed by removing the adaptor sequences, empty reads, and sequences that did not pass the quality threshold. As a result, 148.1 million high-quality reads (83.3%), designated as clean reads, were obtained for both samples (Table S4). By iterative alignment, an average of 99.6% of the clean reads were mapped to the Psv NCPPB 3335 genome, whereas 0.33% of the clean reads did not show any identity with the Psv NCPPB 3335 chromosome or any of its three native plasmids. A summary of the Psv NCPPB 3335 genome coverage obtained with the Illumina RNA-seq data is shown in Table S5. Out of the 5597 coding sequences contained in the Psv NCPPB 3335 chromosome, 5551 genes (99.18%) were covered by Illumina sequencing. Coverage of 99.27% was obtained for the 5596 genes contained in the Psv NCPPB 3335 Δ*hrpL* mutant. In addition, the 68, 53, and 51 genes located in the pPsv48A, pPsv48B, and pPsv48C plasmids, respectively, were also covered by the Illumina RNA sequencing (Table S5).

### 3.3. Characterization of HrpL Regulon of P. savastanoi pv. savastanoi

**Figure 3:**
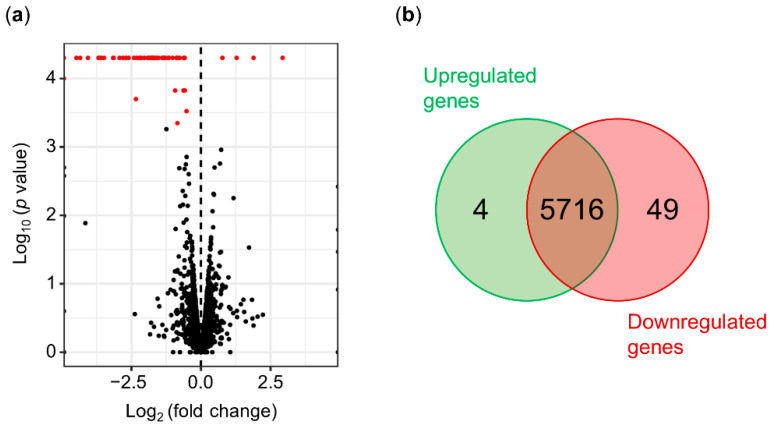
Identification of HrpL-dependent genes in *P. savastanoi* pv. *savastanoi* NCPPB 3335 by RNA-seq. (**a**) Volcano plot showing differentially expressed genes (DEGs) between Psv NCPPB 3335 strain and its Δ*hrpL* mutant. In red, significant DEGs with *q* value < 0.05. (**b**) Venn diagram of significantly upregulated and downregulated genes in Δ*hrpL* mutant relative to wild-type strain in RNA-seq analysis.

**Figure 4:**
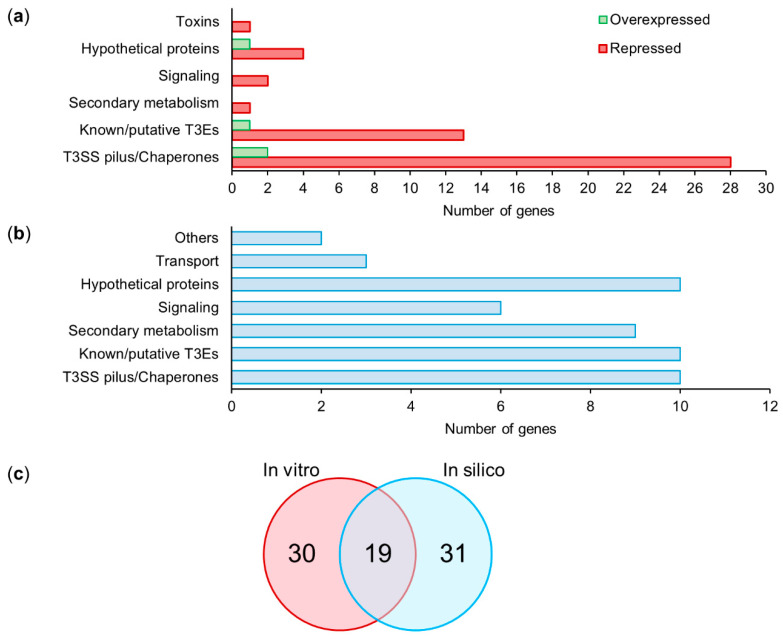
Classification into functional categories of HrpL regulon in *P. savastanoi* pv. *savastanoi* NCPPB 3335. Genes were manually classified into functional categories based on their annotation and/or annotation of encoded proteins showing highest homology in a blastp analysis (https://www.ncbi.nlm.nih.gov/, accessed on 1 June 2021). (**a**) Classification into functional categories of differentially expressed genes (DEGs) identified by RNA-seq. Green and red represent upregulated and downregulated genes, respectively, in Δ*hrpL* mutant relative to wild-type strain. (**b**) Classification into functional categories of bioinformatically predicted genes (BPGs) with putative *hrp*-box in their promoter regions (*hrp* value ≥ 2500). (**c**) Venn diagram of downregulated genes identified by RNA-seq (in vitro) and BPGs (in silico).

After the bioinformatics processing of the raw data, the cleaned reads of each replicate were compared using the cummeRbund package in R [59]. The distribution of normalized FPKM values did not show significant differences among the biological replicates (Figure S2), suggesting that no technical bias was introduced during library construction and sequencing. The normalized expression levels of the wild-type and mutant strains were compared to detect differentially expressed genes (DEGs). The DEGs were selected considering a fold change of ±0.5 and a statistical value of *q* = 0.05 (Figure 3a). A total of 53 DEGs were obtained after the analysis of Psv NCPPB 3335 and its Δ*hrpL* mutant strain. Out of the 53 DEGs, four genes (7.55%) were upregulated, and 49 genes (92.45%) were downregulated relative to the wild-type strain (Figure 3b). Based on their annotation, the DEGs were manually classified into six functional categories (Figure 4a): T3SS pilus/chaperones (30 DEGs), type III effectors (14 DEGs), hypothetical proteins (5 DEGs), signaling (2 DEGs), toxins (1 DEG), and secondary metabolism (1 DEG). 

To obtain a more complete picture of the HrpL regulon in *P. savastanoi*, we performed a bioinformatics search of genes that could putatively be under the regulation of an *hrp*-box. To this end, we used an ad hoc pipeline considering two criteria: the N-terminal sequence features [66], and the presence of potential *hrp*-box 500 nucleotides upstream of the start codon [65]. A total of 50 bioinformatically predicted genes (BPGs) were identified as candidate genes containing a potential *hrp*-box in their promoter regions. Like the DEGs identified by RNA-seq, the BPGs showed a similar functional categorization, except for the toxin category, which was absent, and the presence of transporters and “others” as additional categories (Figure 4b). Similar to the DEGs, T3SS pilus/chaperones (10 BPGs), type III effectors (10 BPGs), and hypothetical proteins (10 BPGs) were the most represented categories of BPGs. The signaling category, with 6 BPGs, was also significantly represented compared with the two DEGs identified by RNA-seq. Interestingly, a Venn diagram representation shows that only 19 out of the 49 DEGs and 50 BPGs were common to both in vitro and in silico analysis (Figure 4c, Table S6), suggesting that both experimental approaches provide valuable and complementary information. 

### 3.4. HrpL Is a Master Regulator of T3SS and Its Effectors in P. savastanoi pv. savastanoi

**Figure 5:**
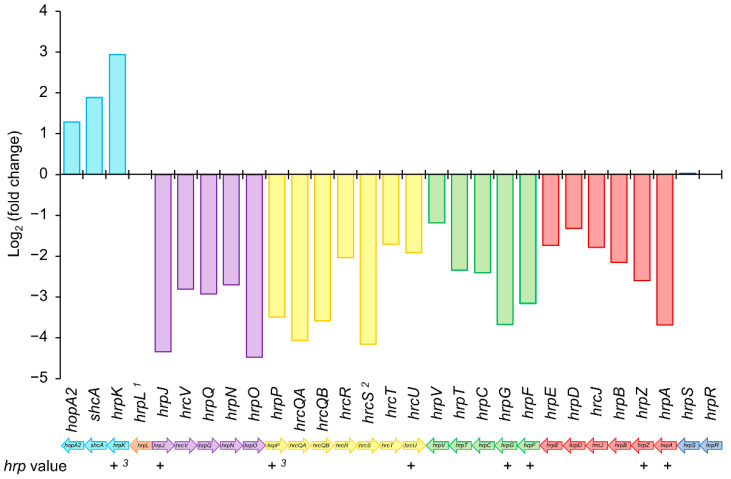
Regulation by HrpL of *hrp*/*hrc* cluster in *P. savastanoi* pv. *savastanoi* NCPPB 3335. Colored bars indicate log_2_ (fold change) value obtained in RNA-seq analysis for wild-type strain vs. its Δ*hrpL* mutant. Negative and positive values indicate genes repressed and overexpressed, respectively, in Δ*hrpL* mutant compared to wild-type strain. ^1^ Expression value obtained for *hrpL* was zero in Δ*hrpL* mutant. ^2^ *q* value < 0.05 was not observed for *hrcS* gene. + and −, presence or absence of *hrp*-box identified bioinformatically. ^3^ *hrp-*box manually identified. Genes (bars) with the same color take part in the same operon.

The *hrp*/*hrc* cluster of Psv NCPPB 3335 is formed by 29 genes organized in five operons (Figure 5), four of which contain structural genes with roles in the secretory system and one formed by *hrpR* and *hrpS*, two regulatory elements that regulate the expression of *hrpL*. Besides these structural and regulatory genes, Psv NCPPB 3335 encodes 31 T3Es in its genome [44,46,47]. Except for the operon formed by *hrpR*/*hrpS*, the remaining structural operons contained an *hrp*-box upstream of their first ORF, suggesting that gene expression is regulated by HrpL. In fact, the RNA-seq data showed that, except for *hrpK* and *hrcS*, the expression of *hrp*/*hrc* genes was downregulated in the Δ*hrpL* mutant compared with the wild-type strain (Figure 5 and Table S7). Although the expression of *hrcS*, a member of the *hrpP* operon, did not show a significant *q* value (0,921), its expression was also downregulated, with a fold change value (log_2_) of −4.15 (Figure 5). However, unlike what was previously described for other strains of the *P. syringae* complex, *hrpK* was significantly overexpressed in the mutant strain compared with the wild-type (Figure 5 and Table S7). Besides *hrpK*, the first gene of the operon (Figure 5), the expression of *schA* and the T3E *hopA2* was also upregulated in the Δ*hrpL* mutant.

**Figure 6:**
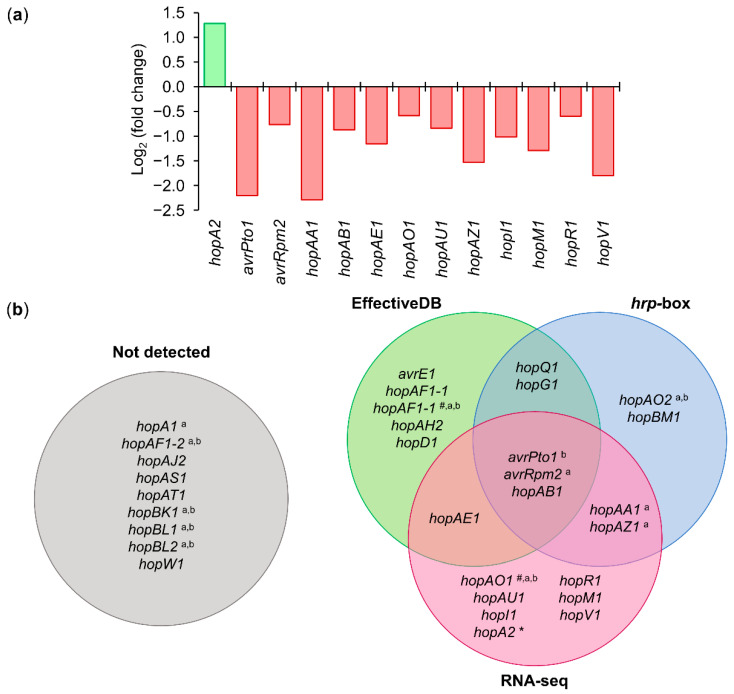
T3Es regulated by HrpL in *P. savastanoi* pv. *savastanoi* NCPPB 3335. (**a**) Graphical representation of log_2_ (fold change) values of T3E genes obtained by RNA-seq analysis of Δ*hrpL* mutant and wild-type strain. Negative and positive values represent downregulation and upregulation of genes, respectively. (**b**) Venn diagram of T3E genes identified by RNA-seq analysis, bioinformatic prediction of *hrp*-box, and N-terminal T3SS targeting pattern (EffectiveDB). Diagram labeled “not detected” represents all T3E genes of Psv NCPPB 3335 that were not identified by the three methods. ^#^ plasmid-encoded genes; ^a^ T3E genes of Psv NCPPB 3335 for which translocation through T3SS was experimentally demonstrated; ^b^ T3E genes for which dependence on HrpL was demonstrated by RT-qPCR in Psv NCPPB 3335 [47,70,71]. * HrpL represses expression of HopA2.

In addition to the structural genes encoded in the *hrp*/*hrc* cluster, RNA-seq analysis showed that the expression of five auxiliary genes of the T3SS, encoding two chaperones (*shcV* and *shcF*) and three helper proteins (*hopAK1*, *hrpW1*, and *hrpH*), was repressed in the Δ*hrpL* mutant (Table 1). Out of these genes, *shcF* formed an operon with *avrRpm2*, a T3E whose expression was also downregulated in the Δ*hrpL* mutant compared with the wild-type strain (Figure 6, Table S8). The bioinformatic prediction showed the presence of an *hrp*-box in three out of the five genes (*hopAK1*, *shcV*, and *shcF*), and for the gene encoding the chaperone ShcM, which forms an operon with *hopM1*, a gene was also identified through RNA-seq analysis (Table 1).

Among the 31 annotated T3Es in Psv NCPPB 3335 [44], RNA-seq analysis identified 12 effectors whose expression was downregulated (*avrPto1, avrRpm2, hopAA1, hopAB1, hopAE1, hopAO1, hopAU1, hopAZ1, hopI1, hopM1, hopR1*, and *hopV1*) and one (*hopA2*) whose expression was upregulated in the Δ*hrpL* mutant compared with the wild-type strain (Figure 6a and Table S8). Among these 13 deregulated genes, two (*avrPto1* and *hopAO1*) coincided with some of the eight genes whose HrpL dependency was demonstrated by RT-qPCR in *P. savastanoi* pv. *savastanoi* NCPPB 3335 [47,70,71]. The bioinformatic analysis predicted a total of 9 genes that contained an *hrp*-box and 11 genes that encoded proteins containing an N-terminal translocation signal (Figure 6b and Table S8). Among the effectors identified by the aforementioned methods, only *avrPto1, avrRpm2*, and *hopAB1* were identified with all of them (Figure 6b). Interestingly, using both of these criteria (RNA-seq analysis and bioinformatic prediction), we identified a putative non-annotated T3E homologous to the XopAD effector from *Xanthomonas*, which is also encoded by *P. syringae* pv. *phaseolicola* 1448A and for which HrpL dependency has been demonstrated (Table S8) [72].

### 3.5. HrpL Regulon of P. savastanoi pv. savastanoi Also Regulates Expression of Genes Unnrelated to T3SS

In addition to genes related to the T3SS, genes encoding proteins annotated as hypothetical proteins, toxins, signaling proteins, and proteins related to secondary metabolites were also identified in the RNA-seq analysis (Table 2). Four out of the five genes encoding hypothetical proteins showed downregulated expression and only one was upregulated in the Δ*hrpL* mutant (Table 2). Among these five genes, the bioinformatic analysis revealed the existence of an *hrp*-box upstream of the *HP02555* and *HP29775* genes and a translocation signal in the protein encoded by the *HP07405* gene. Interestingly, the first gene (*hsvA*) of an operon related to the synthesis of phevamine A, a toxin that can suppress the plant immune response [73], was identified by RNA-seq as part of the HrpL regulon of Psv (Table 2). The expression of *hsvA* was downregulated in the Δ*hrpL* mutant compared with the wild-type strain. Furthermore, genes encoding a TonB-dependent siderophore receptor (*fecA*), a hypothetical protein homologous to a N-acyl homoserine lactone sintase (*HP05360*) and a FMN transferase (*apbE*), were identified by RNA-seq as part of the HrpL regulon of Psv NCPPB 3335 (Table 2).

Although the number of genes identified by RNA-seq was not high, the bioinformatic analysis revealed 26 additional genes containing a predicted *hrp*-box (Table 3). The proteins encoded by all 26 predicted genes were classified into five categories: secondary metabolism, signaling, hypothetical proteins, transporters, and others. For the eight predicted genes encoding hypothetical proteins, their predicted secondary structures were compared with the structures of other proteins included on the HHPred server [69]. However, matches with other proteins were not found. Among the hypothetical proteins, those encoded by *PSA3335_RS11510* and *PSA3335_RS10865* showed a translocation signal, suggesting that they could be putative T3Es. Among these 26 genes, four showed identity with genes whose dependence on HrpL was previously reported in strains belonging to the *P. syringae* complex (Table 3).

## 4. Discussion

We evaluated the importance of HrpL, an essential transcriptional regulator of virulence functions, in the virulence of five pathovars of *P. savastanoi* (Psv, Psn, Psf, Psr, and Psm) that cause disease in woody hosts (olive, oleander, ash, broom, and dipladenia). Given the inability of some *P. savastanoi* strains to induce HR in tobacco plants, the capacity to induce HR in a non-host plant was assessed in tobacco and tomato plants for all the strains and their corresponding Δ*hrpL* mutants (Figure 1). The Psn *Psn23* strain was previously described to induce HR in tobacco leaves, although the assay was performed in the Burley White cultivar [40], a tobacco cultivar different from that used in this work (Newdel). It is well known that the repertoire of bacterial T3Es, as well as the repertoire of plant resistance genes, can vary among bacterial strains or cultivars, thus conditioning the induction of a characteristic HR [76,77]. Furthermore, the characterization of strains Psn *Psn23* and Psm Ph3 shows that they contain a truncated version of *avrPto1*, which encodes a shorter version of the effector with a C-terminal deletion [39,44]. In this sense, it has been described that point mutations occurring at the C-terminal of AvrPto1 prevent HR formation in tobacco but not in tomato leaves, indicating that this part of the effector could participate in its recognition by the plant resistance protein [78]. During the last decade, it has been reported that bacterial species of the *P. syringae* complex can naturally lack the *hrp*/*hrc* cluster, making them unable to induce HR in non-host plants or compromising their ability to cause disease in their respective hosts [19,20,21]. Genomic analysis carried out for Psv NCPPB 3335, Psn *Psn23*, Psm Ph3, Psf NCPPB 1006, and Psr CECT 4861 confirmed that all of these strains contain a canonical *hrp*/*hrc* cluster [39,44], which allows them to cause disease symptoms in their respective woody hosts (Figure 2). In the *P. syringae* complex, the role of HrpL as a global regulator of virulence has been mainly studied in pathogenic bacteria affecting herbaceous plants [48,49,79,80,81]. Our results demonstrate that HrpL is fully required for symptom development in *P. savastanoi* pathovars that cause disease in woody hosts (Figure 2).

The HrpL regulon of *P. syringae* strains, which affect herbaceous plants, has been widely studied using high-throughput sequencing and/or computational analysis [48,49,79,80,81]. In our work, using a combination of both strategies, we identified 53 deregulated genes and 50 candidate genes containing a putative *hrp*-box (Figure 3 and Table 3). Out of the 53 genes identified through RNA-seq analysis, 49 were downregulated compared with the wild-type strain (Figure 3). Analysis carried out with six isolates of *P. syringae* (pv. *phaseolicola*, pv. *lachrymans*, pv. *syringae*, pv. *japonica*, and pv. *tomato*) showed that genes differentially expressed across the strains were mostly upregulated [48]. However, these transcriptomic analyses were performed with mutants transformed with the native *hrpL* gene cloned downstream of an arabinose-inducible promoter. By contrast, our RNA-seq data resulted from a Δ*hrpL* mutant in which the *hrpL* locus was replaced, thus representing the opposite situation reported by Mucyn and associates with overexpressing *P. syringae* strains. In agreement with previous data [48,49], most of the genes identified in our RNA-seq analysis, 44 out of the 53 deregulated genes, were annotated as T3SS components (30 genes) or annotated T3Es (14 genes) (Figure 4), confirming that HrpL mainly regulates the T3SS and its effectors. Unexpectedly, *hrcS* and *hrpK* were two unique genes belonging to the *hrp*/*hrc* cluster that were not significantly deregulated in the Psv Δ*hrpL* mutant. Regarding *hrcS,* while the log_2_ (fold change) value for this gene was about −4.15 (Table S7), suggesting clear repression in the Δ*hrpL* mutant, the *q* value was greater than 0.05, indicating a nonsignificant change. However, the fact that *hrcS* occupies the fifth position in an operon (Figure 5), where all of the upstream (*hrpP*, *hrcQA*, *hrcQB*, and *hrcR*) and downstream (*hrcT* and *hrcU*) genes were significantly repressed, would suggest that differences in transcript abundance across the biological replicates might explain the nonsignificant differences observed for this gene. In relation to *hrpK*, it was previously reported that this gene is activated by HrpL in *P. syringae* strains [48,49]. However, our transcriptomic analysis shows that the *hrpK* gene was significantly upregulated in the Δ*hrpL* mutant compared with its wild-type strain (Table S7). In *P. syringae* pv. *tomato* (Pto) DC3000, it has been described that HrpK is translocated extracellularly through the T3SS, and is required for symptom development and bacterial multiplication [82]. Furthermore, complementation assays for the *hrpK* mutants showed that HrpK does not function inside the plant cells, which would suggest a putative role for HrpK as a T3E translocator located at the plant membrane [82]. In Pto DC3000, *hrpK* is the first gene of an operon together with *hopB1* [83]. However, in Psv NCPPB 3335, this operon is completed by *shcA* (PSA3335_RS10690) and *hopA2* (*hopPsyB*; PSA3335_RS10695), the same operon organization described in *P. syringae* pv. *syringae* 61 [83]. In accordance with the expression data obtained for *hrpK*, the expression of *shcA* and *hopA2* was significantly upregulated in the Δ*hrpL* mutant compared with the wild-type strain (Table 1, Tables S7 and S8). This result suggests that this operon could be repressed, directly or indirectly, by the transcription factor HrpL.

The genomic analysis of Psv NCPPB 3335 revealed that this pathogenic bacterium contains a total of 31 T3Es encoded in its genome [44]. In this study, we identified a total of 16 genes as part of the HrpL regulon, 7 genes identified by RNA-seq, 4 genes predicted bioinformatically, and 5 genes identified by both approaches (Figure 6 and Table S8). Of special interest was the identification of the pseudogene PSA3335_RS02560, which encodes a protein homologous to XopAD, a T3E from *Xanthomonas* [84,85,86]. Strikingly, in the genome of Psv NCPPB 3335, this gene appears to be separated into two ORFs, the pseudogenes PSA3335_RS02560 and PSA3335_RS02565. However, even when both pseudogenes encode proteins that contain the characteristic SKWP repetitions [87], only the expression of PSA3335_RS02560 was found to be repressed in the RNA-seq analysis (Table S8). The fact that PSA3335_RS02560 contains an *hrp*-box within its promoter, as well as the existence of a type III translocation signal at the N-terminal of the protein sequence, suggest that PSA3335_RS02560 and its encoded protein could be a novel T3E acquired by Psv NCPPB 3335 (Table S8). The PSA3335_RS02560 gene is homologous to the PSPPH_1525 gene from *Pseudomonas syringae* pv. *phaseolicola* 1448A, whose expression has been reported to be regulated by HrpL and HrpS [72]. Demonstrating that PSA3335_RS02560 encodes a novel functional T3E in Psv NCPPB 3335 would require additional translocation assays with the protein effector.

An additional goal of work aimed at characterizing the HrpL regulon has been to identify genes, or *hrp*-boxes upstream of the genes, encoding potential virulence factors that are not related to the T3SS [48,49,79,88]. In this study, we identified a total of 34 potential genes whose expression is potentially influenced by HrpL that were not annotated as structural, auxiliary, or functional elements of the T3SS (Figure 4). As previously described, most of these nonrelated T3SS genes were annotated as hypothetical proteins, secondary metabolism, signaling, or toxins (Figure 4 and Table 2 and Table 3). Included in the signaling category, we identified two genes, PSA3335_RS09570 and PSA3335_RS05360, whose expression is regulated by HrpL. The former encodes a TonB-dependent siderophore receptor (FecA) protein (Table 2), an outer membrane transporter involved in citrate-mediated iron import in bacteria [89,90]. The analysis of iron-deficient mutants in *P. syringae* pv. *tomato* DC3000 suggests that, in contrast to mammalian pathosystems, siderophores may play a more specialized role in iron nutrition, growth, and virulence in plant pathogenic bacteria during host colonization [89]. The latter gene, PSA3335_RS05360, was characterized as encoding a hypothetical protein homologous to N-acyl homoserine lactone (AHL) synthase (Table 2). Broadly speaking, AHL is a signaling molecule involved in bacterial quorum sensing (QS), a relevant interbacterial communication system used to detect and respond to cell population density by gene regulation [91,92,93]. In previous work, it was reported that Psv and *Erwinia toletana* share QS signals and cooperate with each other, causing more severe symptoms in olive tree knots [94]. The existence of AHL synthase regulated by HrpL suggests a connection between the T3SS and the low bacterial cell density that exists during the first stages of host colonization.

Bacterial phytotoxins are widely distributed among all the major genera of plant pathogenic bacteria and are considered among the most important virulence factors in symptoms of disease [95,96]. In *P. syringae*, it was previously reported that genes encoding enzymes that participate in the synthesis of coronatine and siringomycin are regulated by HrpL [65,97]. A similar HrpL dependency was observed for the *hrp* PAI of *E. amylovora,* which encodes the proteins required for the synthesis of a putative phaseolotoxin-like phytotoxin [98]. Interestingly, as part of the Psv NCPPB 3335 HrpL regulon, we identified the *hsvA* gene, whose expression was downregulated in the Δ*hrpL* mutant compared with the wild-type strain (Table 2). This gene occupies the first position of the *hsv* operon (*hsvA*, *hsvB*, and *hsvC*), a group of genes involved in synthesizing phevamine A, a small molecule classified as a toxin that suppresses the plant immune response during *P. syringae* infection [73]. It has also been reported that the expression of the *hsvA* operon in *E. amylovora* depends on the HrpL regulator and is required for full virulence in apples [98]. Therefore, characterizing this operon in Psv NCPPB 3335, as well as other *P. savastanoi* strains, would contribute to a better understanding of its role in the virulence of plant pathogenic bacteria infecting woody hosts.

To summarize, high-throughput transcriptome sequencing (RNA-seq) combined with the bioinformatic analysis of sequences containing an *hrp*-box allowed us to characterize the HrpL regulon of *P. savastanoi* pv. *savastanoi* NCPPB 3335. Although most of the identified predicted genes are related to structural or auxiliary functions of the T3SS, the combination of these two experimental approaches showed some potential genes, whose regulation is controlled by HrpL, that could be involved in the virulence of this pathogenic bacterium of woody hosts. Of particular interest was the detection of the *hsvA* gene, which occupies the first position of an operon dedicated to the synthesis of phevamine A, a phytotoxic molecule that participates in the suppression of the plant immune response. The results obtained in this work suggest that HrpL not only orchestrates the expression of genes related to the T3SS but also genes that encode potential virulence factors required for the virulence of *P. savastanoi* in both host and non-host plants. 

## Figures and Tables

**Table 1:** Genes associated with T3SS under regulation of HrpL in *P. savastanoi* pv. *savastanoi* NCPPB 3335.

Accession Number ^a^	Gene	O ^b^	RNA-seq ^c^			HrpL Dependence in other Pathovars ^e^					
			*q* Value	Log_2_ (fc)	*hrp* Value ^d^	Pto	Pph	Psy	Pla	Pja	Por
**Genes Associated with T3SS**											
PSA3335_RS07245	*hopAK1*		0.006	−1.85	2741						
PSA3335_RS10495	*hrpW1*		0.006	−1.66	nd						
PSA3335_RS10540	*hrpH*		0.006	−1.60	nd						
PSA3335_RS10690	*shcA*	2	0.006	1.89							
PSA3335_RS15920	*shcV*		0.006	−3.14	2701						
PSA3335_RS25240	*shcF*	1	0.006	−1.33	2699						
PSA3335_RS10520	*shcM*	1	-	-	2685						

^a^ Accession number in NCBI (https://www.ncbi.nlm.nih.gov/, accessed on 1 June 2021). ^b^ O, operon. Number indicates order of gene within the operon (from 5′ to 3′). ^c^ Genes identified by RNA-seq. Significantly differentially expressed genes have *q* values < 0.05. Fold change (fc) refers to average expression rate obtained in Δ*hrpL* mutant relative to wild-type strain in two biological replicates. Negative (red) and positive (green) log_2_ (fc) correspond to genes downregulated and upregulated, respectively, in Δ*hrpL* mutant relative to wild-type strain. ^d^ Bioinformatic prediction of putative *hrp*-box upstream of start codon of genes encoded in *P. savastanoi* pv. *savastanoi* NCPPB 3335 genome (*hrp* value ≥ 2500); nd, not detected. ^e^ Red box, strains in which activation by HrpL of a homologous protein was identified by RNA-seq [48,49]. Pto, Pph, Psy, Pla, Pja, and Por: *P. syringae* pv. *tomato* DC3000, *P. syringae* pv. *phaseolicola* 1448A, *P. syringae* pv. *syringae* B728A, *P. syringae* pv. *lachrymans* 107, *P. syringae* pv. *japonica* MAFF 301072, and *P. syringae* pv. *oryzae* 1_6, respectively.

**Table 2:** HrpL regulon proteins unrelated to T3SS and identified by RNA-seq in *P. savastanoi* pv. *savastanoi* NCPPB 3335.

Accession Number ^a^	Annotation	Size (aa)	Domains ^b^		Gene ^c^	RNA-seq ^d^		*hrp* Value ^e^	Effective DB ^f^	HrpL Dependence in other Pathovars ^g^					
			Pfam	HHPred		*q* Value	Log_2_ (fc)			Pto	Pph	Psy	Pla	Pja	Por
**Hypothetical proteins**															
PSA3335_RS02555	Hypothetical protein	61	-	-	*HP02555*	0.006	−1.39	2789	nd						
PSA3335_RS07405	Hypothetical protein	41	-	-	*HP07405*	0.012	#	nd	0.9999						
PSA3335_RS29775	Hypothetical protein	40	-	-	*HP29775*	0.006	−2.16	2785	nd						
PSA3335_RS25230	Hypothetical protein	221	-	-	*HP25230*	0.017	−0.58	nd	nd						
PSA3335_RS28495	Hypothetical protein	260	-	-	*HP28495*	0.006	0.77	nd	nd						
**Toxins**															
PSA3335_RS07880	Amidinotransferase	366	-	HsvA protein	*hsvA* ^1^	0.006	−0.87	nd	nd						
PSA3335_RS07885	HPr kinase	418	-	-	*hsvB* ^2^		NS	nd	nd						
PSA3335_RS07890	Hypothetical protein	414	-	-	*hsvC* ^3^		NS	nd	nd						
**Signaling**															
PSA3335_RS09570	TonB-dependent siderophore receptor	782	PF07660 PF07715 PF00593	Iron (III) dicitrate transport protein FecA	*fecA*	0.006	−0.62	nd	nd						
PSA3335_RS05360	Hypothetical protein	212	-	N-acyl homoserine lactone synthase	*HP05360*	0.006	−1.34	2763	nd						
**Secondary metabolism**															
PSA3335_RS20310	FMN transferase	343	PF02424	FMN transferase	*apbE*	0.017	−0.63	2783	nd						

^a^ Accession number in NCBI (https://www.ncbi.nlm.nih.gov/, accessed on 1 June 2021). ^b^ Identification of functional domains in Pfam database [68] and HHPred database [69]. ^c^ 1, 2, and 3 indicate positions (from 5′ to 3′) of genes encoding phevamine A operon. ^d^ Genes identified by RNA-seq. Significantly differentially expressed genes are those with *q* values < 0.05; NS, not significant. Fold change (fc) refers to average expression rate obtained in Δ*hrpL* mutant relative to wild-type strain in two biological replicates. Negative (red) and positive (green) fc correspond to genes downregulated and upregulated, respectively, in Δ*hrpL* mutant relative to wild-type strain. #, indicates that the expression in the Δ*hrpL* mutant is zero. ^e^ Bioinformatic prediction of putative *hrp*-box upstream of start codon of genes encoded in *P. savastanoi* pv. *savastanoi* (Psv) NCPPB 3335 genome (*hrp* value ≥ 2500); nd, not detected. ^f^ EffectiveDB [66] provides values (0.999–1) for proteins in which N-terminal T3SS targeting pattern is detected; nd, not detected. ^g^ Strains in which HrpL dependence of a protein homologous to that identified in Psv NCPPB 3335 was identified by RNA-seq [48,49]. Pto, Pph, Psy, Pla, Pja, and Por: *Pseudomonas syringae* pv. *tomato* DC3000, *P. syringae* pv. *phaseolicola* 1448A, *P. syringae* pv. *syringae* B728A, *P. syringae* pv. *lachrymans* 107, *P. syringae* pv. *japonica* MAFF 301072, and *P. syringae* pv. *oryzae* 1_6, respectively.

**Table 3:** Bioinformatic prediction of putative *hrp*-box upstream of start codon of genes encoded in *P. savastanoi* pv. *savastanoi* NCPPB 3335 genome.

Accession Number ^a^	Annotation	Size (aa)	O ^b^	Place ^b^	*hrp* Value ^c^	EffectiveDB ^d^	Pfam ^e^	HHPred ^f^	HrpL Dependence in other Pathovars ^g^					
									Pto	Pph	Psy	Pla	Pja	Por
**Secondary metabolism**														
PSA3335_RS07710#	Alcohol dehydrogenase	85	yes	1	2718	nd	PF08240	-						
PSA3335_RS12335	Alcohol dehydrogenase	389	yes	2	2624	nd	PF00465	-						
PSA3335_RS26215	1-acyl-sn-glycerol-3-phosphate acyltransferase	270	yes	2	2586	nd	PF01553	-						
PSA3335_RS28260	*iaaL*Psv	397	yes	2	2571	nd	-	-						
PSA3335_RS05405	*iaaL*Psn	395	no	-	2568	nd	-	-						
PSA3335_RS27660	Fructose-1,6-bisphosphate aldolase	354	no	-	2539	nd	PF01116	-						
PSA3335_RS18565	Aminomethyltransferase (*soxA*)	968	yes	5	2511	nd	PF01571 PF17806 PF13510 PF08669 PF07992	-						
PSA3335_RS18560	Sarcosine oxidase subunit delta (*soxD*)	101	yes	4	2511	nd	PF04267	-						
**Signaling**														
PSA3335_RS04530	TetR family transcriptional regulator	220	yes	1	2587	nd	PF08362 PF00440	-						
PSA3335_RS04245	Transcriptional regulator	80	no	-	2572	nd	PF13560							
PSA3335_RS20160	Response regulator transcription factor (*luxR*)	222	yes	2	2570	nd	PF00072 PF00196							
PSA3335_RS25950	Response regulator	121	yes	2	2504	nd	PF00072							
PSA3335_RS07965	Chemotaxis response regulator protein-glutamate methylesterase	358	yes	9	2500	nd	PF01339 PF00072							
**Hypothetical proteins**														
PSA3335_RS00675	Hypothetical protein	224	yes	2	2580	nd	-	Haloacid dehalogenase						
PSA3335_RS11510	Hypothetical protein	289	yes	2	2577	0.9994	PF01904	-						
PSA3335_RS23410#	Hypothetical protein	48	yes	2	2567	nd	PF07551	-						
PSA3335_RS24280#	Hypothetical protein	36	no	-	2542	nd	-	-						
PSA3335_RS13135	Hypothetical protein	79	no	-	2539	nd	-	-						
PSA3335_RS26045	Hypothetical protein	107	yes	5	2531	nd	PF11872	-						
PSA3335_RS06145	Hypothetical protein	166	yes	2	2518	nd	PF14113	T6SS effector Tae4						
PSA3335_RS10865	Hypothetical protein	290	yes	1	2517	0.9998	-	T7 capsid protein						
Transporters														
PSA3335_RS07870	MFS transporter	395	no	-	2560	nd	PF07690							
PSA3335_RS09775	Amino acid ABC transporter permease	365	yes	3	2528	nd	PF00528							
PSA3335_RS20980	MFS transporter	399	no	-	2523	nd	PF07690							
Others														
PSA3335_RS15760	Peptidyl-propyl cis-trans isomerase	91	no	-	2506	nd	PF00639							
PSA3335_RS01395	DUF2628 domain-containing protein	141	no	-	2501	nd	PF10947							

^a^ Accession number in NCBI. # pseudogene. ^b^ Indicates whether identified genes are part of an operon or not and their place within the operon. Prediction of operons was carried out with the fgenesb annotator in SoftBerry [74] and the Operon-mapper web server [75].^c^ Bioinformatic prediction of putative *hrp*-box upstream of start codon of genes encoded in *P. savastanoi* pv. *savastanoi* (Psv) NCPPB 3335 genome (*hrp* value ≥ 2500); nd, not detected. ^d^ EffectiveDB [66] provides values (0.999–1) for proteins in which N-terminal T3SS targeting pattern is detected; nd, not detected. ^e^ Identification of functional domains in Pfam database [68]. ^f^ Identification of structural homologues using HHPred web server [69]. Only results with probability greater than 90% are shown. T6SS, type VI secretion system. ^g^ Red box, strains in which activation by HrpL of a homologous protein was identified by RNA-seq [48,49]. Pto, Pph, Psy, Pla, Pja, and Por: *Pseudomonas syringae* pv. *tomato* DC3000, *P. syringae* pv. *phaseolicola* 1448A, *P. syringae* pv. *syringae* B728A, *P. syringae* pv. *lachrymans* 107, *P. syringae* pv. *japonica* MAFF 301072, and *P. syringae* pv. *oryzae* 1_6, respectively.

## Data Availability

The names of the repository/repositories and accession number(s) can be found in the paper and Supplementary Materials.

## Supplementary Materials

The following are available online at https://www.mdpi.com/article/10.3390/microorganisms9071447/s1. Figure S1: HrpL-dependent expression of HrpL regulon genes by RT-qPCR. Figure S2: Gene expression analysis of biological replicates used in RNA-seq study. Table S1: Strains used in this study. Table S2: Plasmids used in this study. Table S3: List and application of primers used in this work. Table S4: Reads obtained in RNA-seq. Table S5: Summary of Illumina RNA-seq data. Table S6: Genes shared by in vitro and in silico analysis. Table S7: Structural genes of T3SS and genes associated with T3SS under regulation of HrpL in *P. savastanoi* pv. *savastanoi* NCPPB 3335. Table S8: T3E genes under regulation of HrpL in *P. savastanoi* pv. *savastanoi* NCPPB 3335.
