# Supplementary material for: HrpL Regulon of Bacterial Pathogen of Woody Host Pseudomonas savastanoi pv. savastanoi NCPPB 3335
Source: Microorganisms. 2021 Jul 5;9(7):1447. doi: 10.3390/microorganisms9071447 (PMC8303149; doi:10.3390/microorganisms9071447)
Supplement: Supplementary file 1 [file microorganisms-09-01447-s001.zip › microorganisms-1274606-supplementary.pdf]

microorganisms

Article

HrpL Regulon of Bacterial Pathogen of Woody Host *Pseudomonas savastanoi* pv. *savastanoi* NCPPB 3335

Alba Moreno-Pérez [1,2], Cayo Ramos [1,2,*] and Luis Rodríguez-Moreno [1,2,*]

1 Área de Genética, Facultad de Ciencias, Campus Teatinos s/n, Universidad de Málaga, E-29010 Málaga, Spain; albamp@uma.es
2 Departamento de Microbiología y Protección de Cultivos, Instituto de Hortofruticultura Subtropical y Mediterránea «La Mayora», Extensión Campus de Teatinos, Universidad de Málaga-Consejo Superior de Investigaciones Científicas (IHSM-UMA-CSIC), 29010 Málaga, Spain
* Correspondence: crr@uma.es (C.R.); lgrodriguez@uma.es (L.R.-M.); Tel.: +34-952-132146 (C.R.); Tel.: +34-952132131 (L.R.-M.)

Supplementary Material

Figure S1. HrpL-dependent expression of HrpL regulon genes by RT-qPCR. Quantitative reverse-transcription polymerase chain reaction with the indicated *P. savastanoi* pv. *savastanoi* NCPPB 3335 genes for its Δ*hrpL* mutant vs. wild-type (WT) strain at 6 h after transfer to Hrp-inducing medium. Fold change (fc) was calculated after normalization using the *gyrA* gene as an internal control. Results represent the means for two biological replicates. Error bars represent the standard deviation. Asterisks indicate a significantly different value from one, established by a T-Student test (P <0.05), which indicate a significant decrease in the expression of these genes in the Δ*hrpL* mutant compared with WT strain.

Figure S2. Gene expression analysis of biological replicates used in RNA-seq study. The boxplot displays distribution of normalized expression values ($\log_{10}$ FPKM) among the two biological replicates of the *P. savastanoi* pv. *savastanoi* NCPPB 3335 and its Δ*hrpL* mutant.

Table S1. Strains used in this study.

Table S2. Plasmids used in this study.

Table S3. List and application of primers used in this work.

Table S4. Reads obtained in RNA-seq.

Table S5. Summary of Illumina RNA-seq data.

Table S6. Genes shared by in vitro and in silico analysis.

Table S7. Structural genes of T3SS and genes associated with T3SS under regulation of HrpL in *P. savastanoi* pv. *savastanoi* NCPPB 3335.

Table S8. T3E genes under regulation of HrpL in *P. savastanoi* pv. *savastanoi* NCPPB 3335.

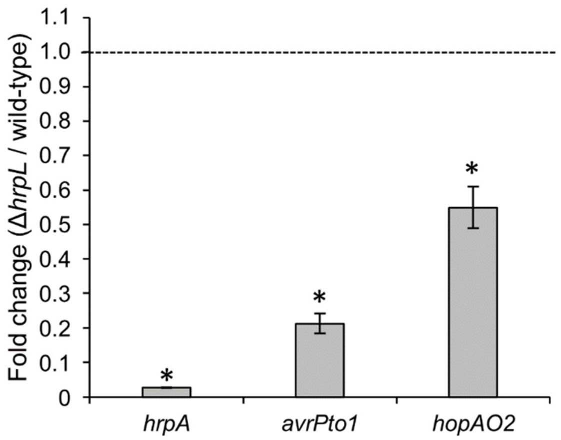

Figure S1. HrpL-dependent expression of HrpL regulon genes by RT-qPCR. Quantitative reverse-transcription polymerase chain reaction with the indicated *P. savastanoi* pv. *savastanoi* NCPPB 3335 genes for its *ΔhrpL* mutant vs. wild-type (WT) strain at 6 h after transfer to Hrp-inducing medium. Fold change (fc) was calculated after normalization using the *gyrA* gene as an internal control. Results represent the means for two biological replicates. Error bars represent the standard deviation. Asterisks indicate a significantly different value from one, established by a T-Student test (P <0.05), which indicate a significant decrease in the expression of these genes in the Δ*hrpL* mutant compared with WT strain.

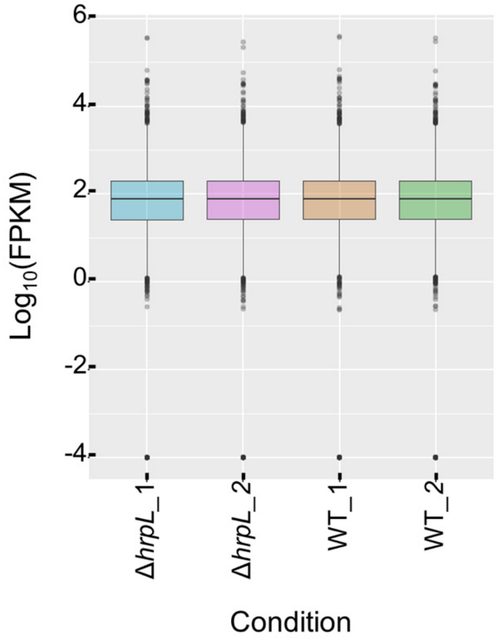

Figure S2. Gene expression analysis of biological replicates used in RNA-seq study. The boxplot displays distribution of normalized expression values ($\log_{10}$ FPKM) among the two biological replicates of *P. savastanoi* pv. *savastanoi* NCPPB 3335 and its Δ*hrpL* mutant.

Table S1. Strains used in this study.

Strain	Main features	Reference or source
Escherichia coli		
DH5α	*F -, ϕ80dlacZ M15, (lacZYA-argF) U169, deoR, recA1, endA, hsdR17 (rk - mk -), phoA, supE44, thi-1, gyrA96, relA1*	[1]
GM2929	*F -, ara-14, leuB6, thi-1, tonA31, lacY1, tsx-78, galK2, galT22, glnV44, hisG4, rpsL136, xyl-5,mtl-1, dam13::Tn9, dcm-6, mcrB1, hsdR2, mcrA, recF143* (Sp^R, Cm^R)	[2]
Pseudomonas		
P. savastanoi* pv. *savastanoi		
NCPPB 3335	Wild-type strain isolated from olive	[3]
NCPPB 3335 Δ*hrpL*::*nptII*	*hrpL* mutant derived from NCPPB 3335 with Km resistance *nptII* cassette (Km^R)	[4]
NCPPB 3335 Δ*hrpL*	*hrpL* mutant derived from NCPPB 3335 Δ*hrpL*::*nptII*	This work
P. savastanoi* pv. *nerii		
Psn23 (ESC23)	Wild-type strain isolated from oleander	[5]
Psn23 Δ*hrpL*::*nptII*	*hrpL* mutant derived from *Psn23* with Km resistance *nptII* cassette (Km^R)	This work
Psn23 Δ*hrpL*	*hrpL* mutant derived from *Psn23* Δ*hrpL*::*nptII*	This work
P. savastanoi* pv. *mandevillae		
Ph3 (CFBP 8832)	Wild-type strain isolated from dipladenia	[6]
Ph3 Δ*hrpL*::*nptII*	*hrpL* mutant derived from Ph3 with Km resistance *nptII* cassette (Km^R)	This work
Ph3 Δ*hrpL*	*hrpL* mutant derived from Ph3 Δ*hrpL*::*nptII*	This work
P. savastanoi* pv. *fraxini		
NCPPB 1006	Wild-type strain isolated from ash	[7]
NCPPB 1006 Δ*hrpL*::*nptII*	*hrpL* mutant derived from NCPPB 1006 with Km resistance *nptII* cassette (Km^R)	This work
NCPPB 1006 Δ*hrpL*	*hrpL* mutant derived from NCPPB 1006 Δ*hrpL*::*nptII*	This work
P. savastanoi* pv. *retacarpa		
CECT 4861	Wild-type strain isolated from broom	[8]
CECT 4861 Δ*hrpL*::*nptII*	*hrpL* mutant derived from CECT 4861 with Km resistance *nptII* cassette (Km^R)	This work
CECT 4861 Δ*hrpL*	*hrpL* mutant derived from CECT 4861 Δ*hrpL*::*nptII*	This work
P. syringae* pv. *tomato		
DC3000	Wild-type strain isolated from tomato	[9]
P. syringae* pv. *tabaci		
CFBP 1621	Wild-type strain isolated from tobacco	[10]/W.S. Beach

Sp, spectinomycin; Cm, chloramphenicol; Km, kanamycin.

Table S2. Plasmids used in this study.

Name	Description	Reference
pGEM-T	Cloning vector containing ori f1 and *lacZ* (Ap^R)	(Promega, USA)
pIAC4-Km	pGEM-T derivate, contains approximately 1.2 kb on each side of the *P. savastanoi* pv. *savastanoi* NCPPB 3335 *hrpL* gene (Ap^R, Km^R)	[4]
pFLP2	Contains a Flp recombinase (flipase) gene and *sacB* (Ap^R)	[11]

Ap, ampicillin; Km, kanamycin.

Table S3. List and application of primers used in this work.

Purpose	Name	Sequence (5′⟶3′)
hrpL probe [a]	*hrpL* F-29	CCTAGTGATCCTTGATGC
	hrpL R-381	CAAGCAATCAATGGCTGC
nptII probe	*nptII* F	GTGTAGGCTGGAGCTGCTTC
	nptII R	TTGCATCAGCCATGATGG
Checking for genomic cleanliness of the RNA	*catB* R1004	GCTCGACCAGAATGTCTTCG
	catB F516	CAAGCTGAAGATCGGTGCC
Real time quantitative PCR	*gyrA* F-180	GAGCGAGCTGGGTAACGACTGGAAC
	gyrA R-329	CAGCGTTGTCGCCATCGACCGAA
	hrpA F-93	ATCAACAGCGTCAAGAGCAG
	hrpA R-267	ATTTGGTTGGAAAGGGCTTC
	avrPto F-295	GACAGGAGAGTCAGGAGTAAACC
	avrPto R-430	AGATCCGCTTGGGTTCATAG
	hopAO2-F	TGCGTTTGATGGTGACCGA
	hopAO2-R	ACCGCAATGGATATGTACCCG

[a] These primers were also used to perform the verification of the possible mutant colonies obtained by PCR.

Table S4. Reads obtained in RNA-seq.

Sample	Raw reads	Clean reads [a]	Reads mapped to reference (%) [b]
**Psv NCPPB 3335**			
Replicate 1	41,289,538	33,083,850 (80.13%)	99.75
Replicate 2	40,704,762	33,925,884 (83.35%)	99.60
Total	81,994,300	67,009,734 (81.72%)	99.67
**Psv NCPPB 3335 Δ*hrpL***			
Replicate 1	47,862,912	40,450,444 (84.51%)	99.67
Replicate 2	47,974,950	40,705,092 (84.85%)	99.65
Total	95,837,862	81,155,536 (84.68%)	99.66
**Total RNA-seq**	177,832,162	148,165,270 (83.32%)	99.67

[a] Clean reads and percentage of accepted reads obtained after preprocessing with SeqTrimNext. [b] Percentage of clean reads that mapped with fully closed reference genome of *P. savastanoi* pv. *savastanoi* NCPPB 3335 including its native plasmids, pPsv48A, pPsv48B, and pPsv48C.

Table S5. Summary of Illumina RNA-seq data.

	**Psv NCPPB 3335**		**Psv NCPPB 3335 Δ*hrpL***	
	Replicate 1	Replicate 2	Replicate 1	Replicate 2
Technology	Illumina	Illumina	Illumina	Illumina

Read length (nt)	75	75	75	75
Phred score (% ≥Q30)	>88%	>88%	>88%	>89%
Genome size (bp)	6,143,577	6,143,577	6,143,022	6,143,022
Sequencing depth[a]	344.2	351.6	420.8	423.5
CDS in chromosome	5597	5597	5596	5596
% CDS covered by reads	99.18	99.18	99.27	99.29
CDS in plasmids	172	172	172	172
% CDS covered by reads	100.00	99.42 [b]	100.00	100.00

[a] Sequencing depth was defined as number of nucleotides used for analysis divided by the genome size plus the three plasmids. [b] A CDS (coding sequence) from plasmid pPsv48C was not covered.

Table S6. Genes shared by in vitro and in silico analysis.

**Accession number [a]**	**Protein ID [a]**	**Length [b]**	**Annotation**	**Gene**
			**Type III secretion system (T3SS)**	
PSA3335_RS10675	WP_002552422.1	368	YopN family type III secretion system gatekeeper subunit	*hrpJ*
PSA3335_RS10620	WP_004666089.1	359	EscU/YscU/HrcU family type III secretion system export apparatus switch protein	*hrcU*
PSA3335_RS10600	WP_002552409.1	67	Hypothetical protein	*hrpT*
PSA3335_RS10590	WP_080393802.1	146	Type III secretion protein	*hrpG*
PSA3335_RS10585	WP_002552406.1	74	Type III secretion protein	*hrpF*
PSA3335_RS10560	WP_002552401.1	347	Type III secretion system helper protein	*hrpZ*
PSA3335_RS10555	WP_002552400.1	108	Membrane protein	*hrpA*
			**Helpers T3SS**	
PSA3335_RS07245	WP_002554797.1	524	Two-domain harpin	*hopAK1*
PSA3335_RS15920	WP_004665222.1	96	Type III chaperone protein ShcV	*ShcV*
PSA3335_RS25240	WP_031597912.1	131	Type III chaperone protein ShcF	*ShcF*
			**T3SS effectors**	
PSA3335_RS01620	WP_002551447.1	162	Hypothetical protein	*avrPto1*
PSA3335_RS25235	WP_002556083.1	223	Hypothetical protein	*avrRpm2*
PSA3335_RS02535	WP_002556043.1	257	Hypothetical protein	*hopAB1*
PSA3335_RS20790	Pseudogene	110	DUF1826 domain-containing protein	*hopAZ1*
PSA3335_RS02560	Pseudogene	1712	Type III effector protein XopAD	*xopAD*
			**Hypothetical proteins**	
PSA3335_RS02555	WP_080719001.1	61	Hypothetical protein	*HP02555*
PSA3335_RS29775	Pseudogene	40	Hypothetical protein	*HP29775*
			**Signaling**	
PSA3335_RS05360	WP_031599113.1	212	Hypothetical protein	*HP05360*
			**Secondary metabolism**	
PSA3335_RS20310	WP_002552885.1	343	FAD: protein FMN transferase	*HP20310*

[a] Accession number and protein ID in NCBI (https://www.ncbi.nlm.nih.gov/). [b] Length in amino acids.

Table S7. Structural genes of T3SS and genes associated with T3SS under regulation of HrpL in *P. savastanoi* pv. *savastanoi* NCPPB 3335.

Accession number [a]	Gene	O [b]	RNA-seq [c]		*hrp* value [d]	HrpL dependence in other pathovars [e]					
			q value	Log_2 (fc)		Pto	Pph	Psy	Pla	Pja	Por
**T3SS pilus**											
PSA3335_RS10685	*hrpK*	1	0.006	2.93	nd	■	■	■	■	■	■
PSA3335_RS10680	*hrpL*	1	0.006	#	nd						
PSA3335_RS10675	*hrpJ*	1	0.006	−4.34	2779	■	■	■	■	■	■
PSA3335_RS10670	*hrcV*	2	0.006	−2.81		■	■	■	■	■	■
PSA3335_RS10665	*hrpQ*	3	0.006	−2.93		■	■	■	■	■	■
PSA3335_RS10660	*hrcN*	4	0.006	−2.70		■	■	■	■	■	■
PSA3335_RS10655	*hrpO*	5	0.006	−4.48		■	■	■	■	■	■
PSA3335_RS10650	*hrpP*	1	0.006	−3.49	nd	■	■	■	■	■	■
PSA3335_RS10645	*hrcQA*	2	0.006	−4.06		■	■	■	■	■	■
PSA3335_RS10640	*hrcQB*	3	0.006	−3.59		■	■	■	■		■
PSA3335_RS10635	*hrcR*	4	0.006	−2.04		■	■	■	■	■	■
PSA3335_RS10630	*hrcS*	5	NS	−4.15		■	■	■	■	■	■
PSA3335_RS10625	*hrcT*	6	0.006	−1.71		■	■	■	■	■	■
PSA3335_RS10620	*hrcU*	6	0.006	−1.92	2585	■	■	■	■	■	■
PSA3335_RS10605	*hrpV*	5	0.006	−1.18		■	■	■	■	■	■
PSA3335_RS10600	*hrpT*	4	0.022	−2.34		■	■	■	■	■	■
PSA3335_RS10595	*hrcC*	3	0.006	−2.40		■	■	■	■	■	■
PSA3335_RS10590	*hrpG*	2	0.006	−3.67	2796	■	■	■	■	■	■
PSA3335_RS10585	*hrpF*	1	0.006	−3.16	2796	■	■	■	■		■
PSA3335_RS10580	*hrpE*	6	0.006	−1.73		■	■	■	■	■	■
PSA3335_RS10575	*hrpD*	5	0.006	−1.32		■	■	■	■	■	■
PSA3335_RS10570	*hrcJ*	4	0.006	−1.79		■	■	■	■	■	■
PSA3335_RS10565	*hrpB*	3	0.006	−2.16		■	■	■	■	■	■
PSA3335_RS10560	*hrpZ*	2	0.006	−2.60	2739	■	■	■	■	■	■
PSA3335_RS10555	*hrpA*	1	0.006	−3.68	2739	■	■	■	■	■	■
PSA3335_RS10550	*hrpS*	2	NS	0.03							
PSA3335_RS10545	*hrpR*	1	NS	0.00							

[a] Accession number in NCBI (https://www.ncbi.nlm.nih.gov/). [b] O, operon. Number indicates order of gene within the operon (from 5′ to 3′). Blue shading indicates first gene of operon and individually transcribed genes. [c] Genes identified by RNA-seq. Significantly differentially expressed genes have *q* values < 0.05; NS, not significant. Fold change (fc) refers to average expression rate obtained in Δ*hrpL* mutant relative to wild-type strain in two biological replicates. Negative (red) and positive (green) log_2 (fc) correspond to genes downregulated and upregulated, respectively, in Δ*hrpL* mutant relative to wild-type strain. #, indicates that the expression in the Δ*hrpL* mutant is zero. [d] Bioinformatic prediction of putative *hrp*-box upstream of start codon of genes encoded in *P. savastanoi* pv. *savastanoi* NCPPB 3335 genome (*hrp* value ≥ 2500); nd, not detected. [e] Red box, strains in which activation by HrpL of a homologous protein was identified by RNA-seq [12,13]. Pto, Pph, Psy, Pla, Pja, and Por: *P. syringae* pv. *tomato* DC3000, *P. syringae* pv. *phaseolicola* 1448A, *P. syringae* pv. *syringae* B728A, *P. syringae* pv. *lachrymans* 107, *P. syringae* pv. *japonica* MAFF 301072, and *P. syringae* pv. *oryzae* 1_6, respectively.

Table S8. T3Es genes under regulation of HrpL in *P. savastanoi* pv. *savastanoi* NCPPB 3335.

Accession number [a]	Gene	RNA-seq [c]		*hrp* value [d]	EffectiveDB [e]	HrpL dependence in other pathovars [f]					
		q value	Log_2 (fc)			Pto	Pph	Psy	Pla	Pja	Por
**Known T3Es**											
PSA3335_RS10535*	*avrE1*		NS	nd	0.999	■	■	■	■	■	■
PSA3335_RS01620	*avrPto1* [b]	0.006	−2.21	2832	1.000	■		■	■		■
PSA3335_RS25235	*avrRpm2*	0.006	−0.77	2699	1.000						
PSA3335_RS05460	*hopA1*		NS	nd	nd	■			■		
PSA3335_RS10695	*hopA2*	0.006	1.28	nd	nd						
PSA3335_RS10490	*hopAA1*	0.006	−2.29	2728	nd	■		■	■	■	■
PSA3335_RS02535	*hopAB1*	0.006	−0.87	2591	0.999		■	■			
PSA3335_RS24240	*hopAE1*	0.006	−1.16	nd	1.000				■		
PSA3335_RS29755*	*hopAF1-1*		NS	nd	1.000						
PSA3335_A0028	*hopAF1-1* [b]		NS	nd	1.000				■		
PSA3335_RS29760*	*hopAF1-2* [b]		NS	nd	nd	■	■	■			■
PSA3335_RS14505	*hopAH2*		NS	nd	1.000						
PSA3335_RS24880	*hopAJ2*		NS	nd	nd						
PSPSV_B0010	*hopAO1* [b]	0.006	−0.59	nd	nd	■			■		■
PSA3335_RS12020	*hopAO2* [b]		NS	2579	nd						
PSA3335_RS02305*	*hopAS1*		NS	nd	nd		■				
Not available	*hopAT1*		NS	nd	nd		■				
PSA3335_RS12045*	*hopAU1*	0.006	−0.84	nd	nd		■				
PSA3335_RS20790*	*hopAZ1*	0.006	−1.53	2770	nd				■		
PSA3335_RS19200	*hopBK1* [b]		NS	nd	nd						
PSA3335_RS02885*	*hopBL1* [b]		NS	nd	nd						
PSA3335_RS03825*	*hopBL2* [b]		NS	nd	nd						
PSA3335_RS02795	*hopBM1*		NS	2546	nd	■					
PSA3335_RS28230	*hopD1*		NS	nd	1.000		■				■
PSA3335_RS21975	*hopG1*		NS	2634	1.000	■	■				■
PSA3335_RS24700	*hopI1*	0.006	−1.02	nd	nd	■	■	■	■	■	
PSA3335_RS10525	*hopM1*	0.006	−1.29	nd	nd	■		■	■	■	■
PSA3335_RS28225	*hopQ1*		NS	2554	0.999	■	■				■
PSA3335_RS28250	*hopR1*	0.006	−0.60	nd	nd	■	■				■
PSA3335_RS15915*	*hopV1*	0.006	−1.80	nd	nd	■	■				■
PSA3335_RS29445	*hopW1*		NS	nd	nd						
**New putativeT3Es**											
PSA3335_RS02560	*xopAD*	0.032	−0.52	2789	1.000		■				

[a] Accession number in NCBI (https://www.ncbi.nlm.nih.gov/). Asterisks indicate genes whose length in NCBI does not match which previously described in HopDatabase (http://www.pseudomonas-syringae.org/). [b] Genes whose HrpL dependency was previously demonstrated by RT-qPCR in *P. savastanoi* pv. *savastanoi* NCPPB 3335 [4,14,15]. [c] Genes identified by RNA-seq. Significantly differentially expressed genes have *q* values < 0.05; NS, not significant. Fold change (fc) refers to average expression rate obtained in

Δ*hrpL* mutant relative to wild-type strain in two biological replicates. Negative (red) and positive (green) log_2 (fc) correspond to genes downregulated and upregulated, respectively, in Δ*hrpL* mutant relative to wild-type strain. [d] Bioinformatic prediction of putative *hrp*-box upstream of start codon of genes encoded in *P. savastanoi* pv. *savastanoi* NCPPB 3335 genome (*hrp* value ⩾ 2500); nd, not detected. [e] EffectiveDB [16] provides values (0.999–1) for proteins in which N-terminal T3SS targeting pattern is detected; nd, not detected. [f] Red box, strains in which activation by HrpL of a homologous protein was identified by RNA-seq [12,13]. Pto, Pph, Psy, Pla, Pja, and Por: *P. syringae* pv. *tomato* DC3000, *P. syringae* pv. *phaseolicola* 1448A, *P. syringae* pv. *syringae* B728A, *P. syringae* pv. *lachrymans* 107, *P. syringae* pv. *japonica* MAFF 301072, and *P. syringae* pv. *oryzae* 1_6, respectively.

References

1. Hanahan, D. Studies on transformation of *Escherichia coli* with plasmids. *J. Mol. Biol.* **1983**, *166*, 557-580, doi:10.1016/s0022-2836(83)80284-8.
2. Bullock, W.O.; Fernandez, J.M.; Short, J.M. Xl1-Blue: a high efficiency plasmid transforming *recA Escherichia coli* strain with beta-galactosidase selection. *Biotechniques* **1987**, *5*, 376-378.
3. Pérez-Martínez, I.; Rodríguez-Moreno, L.; Matas, I.M.; Ramos, C. Strain selection and improvement of gene transfer for genetic manipulation of *Pseudomonas savastanoi* isolated from olive knots. *Res. Microbiol.* **2007**, *158*, 60-69, doi:10.1016/j.resmic.2006.09.008.
4. Matas, I.M.; Castañeda-Ojeda, M.P.; Aragón, I.M.; Antúnez-Lamas, M.; Murillo, J.; Rodríguez-Palenzuela, P.; López-Solanilla, E.; Ramos, C. Translocation and functional analysis of *Pseudomonas savastanoi* pv. savastanoi NCPPB 3335 type III secretion system effectors reveals two novel effector families of the *Pseudomonas syringae* complex. *Mol. Plant-Microbe Interact.* **2014**, *27*, 424-436, doi:10.1094/MPMI-07-13-0206-R.
5. Tegli, S.; Gori, A.; Cerboneschi, M.; Cipriani, M.G.; Sisto, A. Type three secretion system in *Pseudomonas savastanoi* pathovars: does timing matter? *Genes* **2011**, *2*, 957, doi:10.3390/genes2040957.
6. Eltlbany, N.; Prokscha, Z.-Z.; Castañeda-Ojeda, M.P.; Krögerrecklenfort, E.; Heuer, H.; Wohanka, W.; Ramos, C.; Smalla, K. A new bacterial disease on *Mandevilla sanderi*, caused by *Pseudomonas savastanoi*: lessons learned for bacterial diversity studies. *Appl. Environ. Microbiol.* **2012**, *78*, 8492-8497, doi:10.1128/AEM.02049-12.
7. Janse, J.D. The bacterial disease of ash (*Fraxinus excelsior*), caused by *Pseudomonas syringae* subsp. *savastanoi* pv. fraxini. II. Etiology and taxonomic considerations. *European Journal of Forest Pathology* **1981**, *11*, 425-438, doi:10.1111/j.1439-0329.1981.tb00115.x.
8. Álvarez, F.; García de los Ríos, J.E.; Jimenez, P.; Rojas, A.; Reche, P.; Troya, M.T. Phenotypic variability in different strains of *Pseudomonas syringae* subsp. savastanoi isolated from different hosts. *Eur. J. Plant Pathol.* **1998**, *104*, 603-609, doi:10.1023/A:1008604011154.
9. Cuppels, D.A. Generation and characterization of Tn5 insertion mutations in *Pseudomonas syringae* pv. tomato. *Appl. Environ. Microbiol.* **1986**, *51*, 323-327, doi:10.1128/AEM.51.2.323-327.1986.
10. Guillorit, C.; Samson, R. A Note: Serological study of four pathovars of *Pseudomonas syringae*: Ps. syr. aptata, Ps. syr. tabaci, Ps. syr. mors-prunorum and Ps. syr. phaseolicola. *J. Appl. Bacteriol.* **1993**, *74*, 683-687, doi:10.1111/j.1365-2672.1993.tb05203.x.
11. Hoang, T.T.; Karkhoff-Schweizer, R.R.; Kutchma, A.J.; Schweizer, H.P. A broad-host-range Flp-*FRT* recombination system for site-specific excision of chromosomally-located DNA sequences: application for isolation of unmarked *Pseudomonas aeruginosa* mutants. *Gene* **1998**, *212*, 77-86, doi:10.1016/S0378-1119(98)00130-9.
12. Mucyn, T.S.; Yourstone, S.; Lind, A.L.; Biswas, S.; Nishimura, M.T.; Baltrus, D.A.; Cumbie, J.S.; Chang, J.H.; Jones, C.D.; Dangl, J.L.; et al. Variable suites of non-effector genes are co-regulated in the type III secretion virulence regulon across the *Pseudomonas syringae* phylogeny. *PLoS Pathog.* **2014**, *10*, e1003807, doi:10.1371/journal.ppat.1003807.
13. Lam, H.N.; Chakravarthy, S.; Wei, H.L.; BuiNguyen, H.; Stodghill, P.V.; Collmer, A. Global analysis of the HrpL regulon in the plant pathogen *Pseudomonas syringae* pv. tomato DC3000 reveals new regulon members with diverse functions. *PLoS ONE* **2014**, *9*, e106115, doi:10.1371/journal.pone.0106115.

14. Castañeda-Ojeda, M.P.; Moreno-Pérez, A.; Ramos, C.; López-Solanilla, E. Suppression of plant immune responses by the *Pseudomonas savastanoi* pv. savastanoi NCPPB 3335 type III effector tyrosine phosphatases HopAO1 and HopAO2. *Front. Plant Sci.* **2017**, *8*, 680, doi:10.3389/fpls.2017.00680.
15. Castañeda-Ojeda, M.P.; López-Solanilla, E.; Ramos, C. Differential modulation of plant immune responses by diverse members of the *Pseudomonas savastanoi* pv. savastanoi HopAF type III effector family. *Mol. Plant Pathol.* **2017**, *18*, 625-634, doi:10.1111/mpp.12420.
16. Eichinger, V.; Nussbaumer, T.; Platzer, A.; Jehl, M.-A.; Arnold, R.; Rattei, T. EffectiveDB—updates and novel features for a better annotation of bacterial secreted proteins and type III, IV, VI secretion systems. *Nucleic Acids Res.* **2015**, *44*, D669-D674, doi:10.1093/nar/gkv1269.
